# Microwave-Assisted Green Synthesis of Silver Nanoparticles Using *Juglans regia* Leaf Extract and Evaluation of Their Physico-Chemical and Antibacterial Properties

**DOI:** 10.3390/antibiotics7030068

**Published:** 2018-07-30

**Authors:** Mahsa Eshghi, Hamideh Vaghari, Yahya Najian, Mohammad Javad Najian, Hoda Jafarizadeh-Malmiri, Aydin Berenjian

**Affiliations:** 1 Faculty of Chemical Engineering, Sahand University of Technology, Tabriz 513351996, Iran; mahsaeshghii@gmail.com (M.E.); vaghari_h@yahoo.com (H.V.); 2 Research and Development Department, Najian Herbal Group, East Azarbaijan, Tabriz 5159193858, Iran; h_jafarizadeh@yahoo.com (Y.N.); najian_herbal_group@yahoo.com (M.J.N.); 3 School of Engineering, Faculty of Science and Engineering, University of Waikato, Hamilton 3240, New Zealand

**Keywords:** green synthesis, silver nanoparticles, microwave irradiation, *Juglans regia*, antibacterial activity

## Abstract

Silver nanoparticles (Ag NPs) were synthesized using *Juglans regia* (*J. regia*) leaf extract, as both reducing and stabilizing agents through microwave irradiation method. The effects of a 1% (*w*/*v*) amount of leaf extract (0.1–0.9 mL) and an amount of 1 mM AgNO_3_ solution (15–25 mL) on the broad emission peak (λ_max_) and concentration of the synthesized Ag NPs solution were investigated using response surface methodology (RSM). Fourier transform infrared analysis indicated the main functional groups existing in the *J. regia* leaf extract. Dynamic light scattering, UV-Vis spectroscopy and transmission electron microscopy were used to characterize the synthesized Ag NPs. Fabricated Ag NPs with the mean particle size and polydispersity index and maximum concentration and zeta potential of 168 nm, 0.419, 135.16 ppm and −15.6 mV, respectively, were obtained using 0.1 mL of *J. regia* leaf extract and 15 mL of AgNO_3_. The antibacterial activity of the fabricated Ag NPs was assessed against both Gram negative (*Escherichia coli*) and positive (*Staphylococcus aureus*) bacteria and was found to possess high bactericidal effects.

## 1. Introduction

Silver is known as strong antimicrobial agent due to its toxicity against most microorganisms including bacteria, fungi, and viruses [1]. As compared to the silver element in bulk state, silver nanoparticles (Ag NPs) exhibit unusual physico-chemical and antimicrobial properties due to their lower particle size (less than 100 nm) and higher surface area to volume ratio, which increase the proportion of high-energy surface atoms [1,2,3]. Because of the marvelous antimicrobial properties of Ag NPs, they have been widely utilized in numerous fields such as medicine, electronics, biotechnology, water disinfection, air treatment, and packaging [3]. 

Green chemistry is the use of chemistry principles to reduce or eliminate the use of toxic reagents, leading to lower amounts of undesirable residues, which in turn are harmful to human health or the environment [4,5]. Incorporation of green chemistry and nanotechnology is of great interest and has gained much attention over the past decade. Green synthesis of Ag NPs, using plants and their derivative extracts as an alternative method for the conventional physical and chemical NP fabrication methods, has attracted considerable attention in recent years [1,6]. In fact, various metabolites existing in the plants including sugars, alkaloids, phenolic acids, terpenoids, polyphenols, and proteins play an important role in the bioreduction of silver ions to Ag NPs and their stabilization [7]. Several studies have been completed on green synthesis of Ag NPs using *Aloe vera* and *Pelargonium zonali* leaf extract and chitosan [8,9,10].

Walnut belongs to *Juglandaceae* family and has the scientific name *Juglans regia* (*J. regia*). Numerous health benefits of walnut for human have been reported, some of which are related to the existence of its main bioactive compounds including polyphenolic compounds, flavonoids, proteins, carotenoids, lipids, and alkaloids. Leaf of walnut contains malic acid, sucrose, α-tocopherol, 3-*O*-caffeoylquinic acids, and quercetin *O*-pentoside as the most abundant organic acids, disaccharide, tocopherol isomer and phenolic compounds, respectively [11,12,13].

The present work focuses on (i) the potential of walnut leaf extract in the fabrication of Ag NPs; (ii) Ag NPs synthesized parameter optimization using microwave irradiation to achieve Ag NPs with more desirable physico-chemical properties and (iii) an antibacterial activity assessment of the produced Ag NPs. 

## 2. Results

### 2.1. Formation of Ag NPs

**Figure 1:**
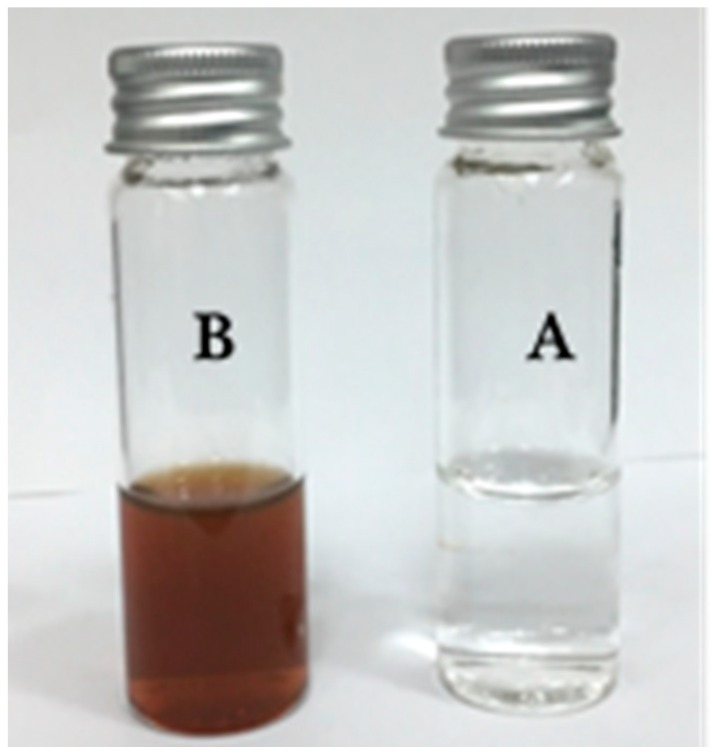
Color and appearance changes during synthesis of silver nanoparticles (Ag NPs) using *J. regia* leaf extract. *J. regia* leaf extract containing silver nitrate before (**A**) and after (**B**) exposure to microwave irradiation.

During the synthesis of Ag NPs, the color of the mixture solution containing silver salt and walnut leaf extract changed from colorless into brown and dark. In fact, the synthesized Ag NPs, due to their surface plasmon resonance (SPR), changed the color of the mixture solution and this color change verified the formation of Ag NPs using walnut leaf extract and microwave irradiation (Figure 1). As clearly observed in Table 1, λ_max_ of the synthesized Ag NPs was varied 424–429 nm, which was in a favorable range for Ag NPs [8,10]. The particle size of the synthesized Ag NPs could be correlated with their broad emission peaks (λ_max_), where longer wavelengths in λ_max_ of Ag NPs were associated with bigger size [9]. This indicated that *J. regia* leaf extract successfully reduced silver ions and formed Ag NPs. 

**Figure 2:**
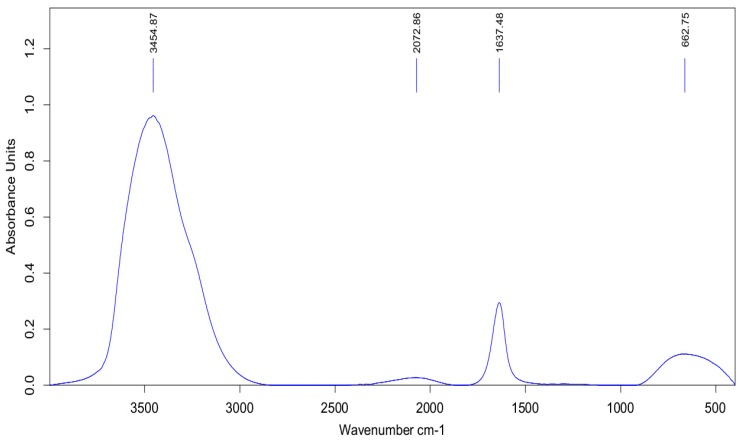
Fourier transform-infrared (FT-IR) spectrum of *J. regia* leaf extract.

Figure 2 shows the Fourier transform-infrared (FT-IR) spectrum of *J. regia* leaf extract which was in the region range of 400–4000 cm^−1^. The IR spectrum of leaf extract absorption bands at 3454.87 and 2072.86 cm^−1^ represent the phenolic–OH and N=C groups. Absorption bands at 1637.48 cm^−1^ are related to the amino group, bending vibrations, and the –OH group. The obtained results indicated that the phenolic compounds and proteins were the two main components of the *J. regia* leaf extract which had key roles in the formation of the stabilized Ag NPs [11,12]. 

### 2.2. Models Generation and Synthesis Conditions Optimization

Based on experimental runs, the values of broad emission peaks (λ_max_, nm) and concentration (ppm) for the fabricated Ag NPs were achieved (Table 1) and according to these obtained data, models were created. Table 2 shows coefficients of the model terms and models accuracy based on R square and its adjusted (R^2^, R^2^-adj) and lack of fit. The high values of R^2^ (>77.80) and R^2^-adj (>85.61) and lack of fit (*p* > 0.05) verified higher suitability of the generated models to predict the synthesis response parameters. *p*-Values of the generated model terms are also described in Table 3. As can be seen, the interaction of the amounts of leaf extract and silver salt solution had significant (*p* < 0.05) effects on λ_max_ and concentration of the formed Ag NPs. 

**Figure 3:**
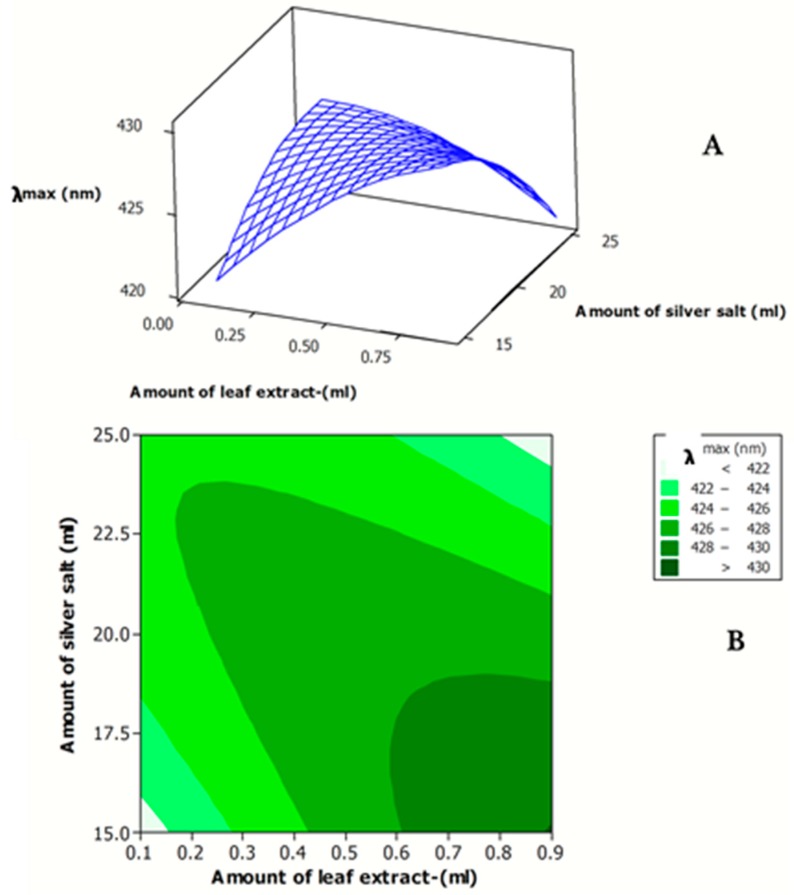
Response surface (**A**) and contour plots (**B**) for λ_max_ of the synthesized Ag NP solution as function of the amount of *J. regia* leaf extract and amount of AgNO_3_ solution.

**Figure 4:**
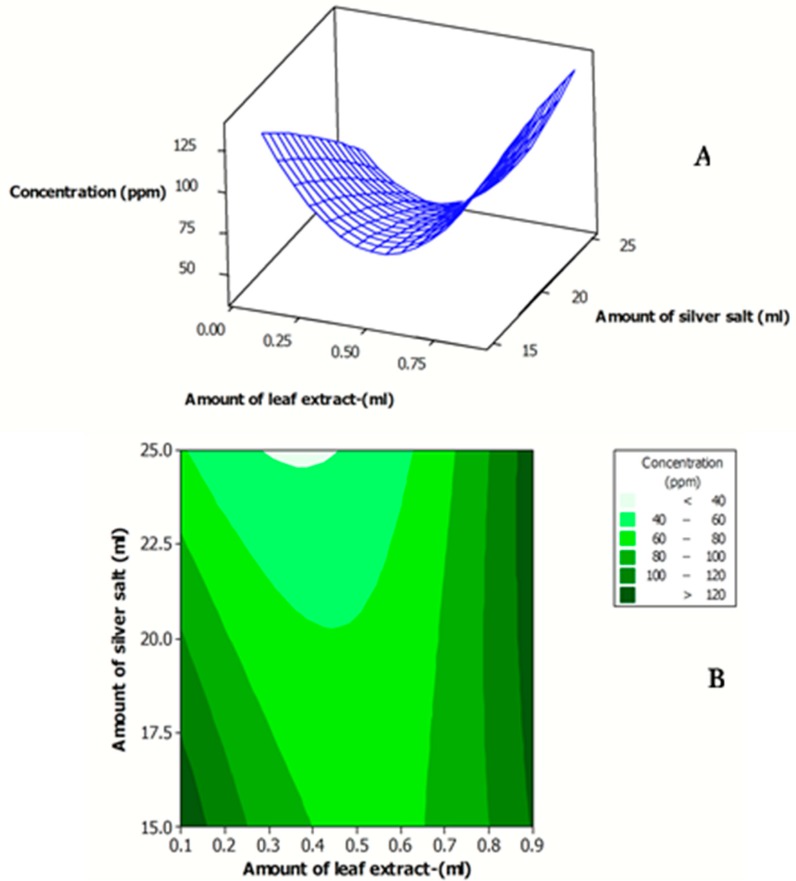
Response surface (**A**) and contour plots (**B**) for the concentration of the synthesized Ag NP solution as function of amount of *J. regia* leaf extract and amount of AgNO_3_ solution.

Figure 3A,B indicates the effects of the amount of *J. regia* leaf extract and amount of AgNO_3_ on the λ_max_ of the synthesized Ag NPs. As clearly observed in Figure 3, the minimum λ_max_ (particle size) was obtained at both minimum amount of *J. regia* leaf extract and of AgNO_3_ solution and the maximum amount of *J. regia* leaf extract and of AgNO_3_ solution. The experimental value of the concentration for the synthesized Ag NPs ranged 19–80 ppm (Table 1). The effects of the amount of *J. regia* leaf extract andof AgNO_3_ on the concentration of the fabricated Ag NPs are shown in Figure 4A,B.

As clearly observed in Figure 4, the maximum concentration of the synthesized Ag NPs was obtained using the highest amount of the *J. regia* leaf extract. The obtained results were in line with the findings of other research [8,9,10]. They found that by increasing the amount of plant extract, the concentration of the bioreductants increased in the extract, which in turn increased the concentration of the formed Ag NPs.

**Figure 5:**
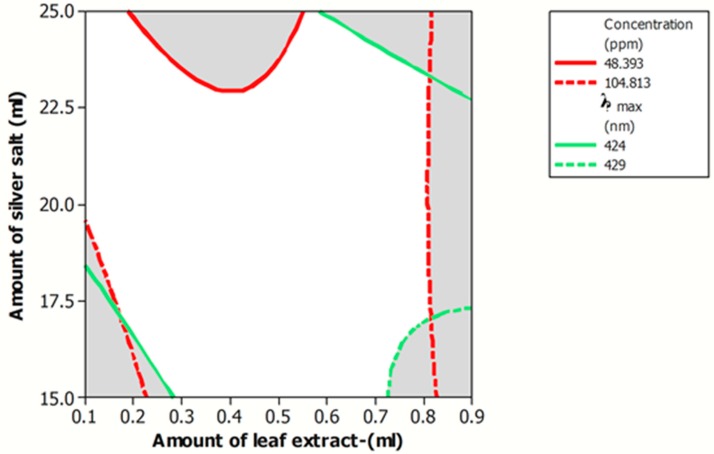
Overlaid contour plot of Ag NPs λ_max_ and concentration with acceptable levels as a function of amount of *J. regia* leaf extract and amount of AgNO_3_ solution.

In the synthesis of Ag NPs, the main objective is formation of NPs with desirable physico-chemical properties, including minimum particle size (λ_max_) and maximum concentration. This synthesis process is known as optimized procedure. According to the generated models for the synthesis of Ag NPs, an overlaid contour plot, as graphical optimization (Figure 5), was plotted to better visualize of optimum area (white colored zone). 

The result of a numerical optimization also demonstrated that by using 0.1 mL of *J. regia* leaf extract and 15 mL of AgNO_3_, Ag NPs with minimum λ_max_ of 421 nm and maximum concentration of 135.64 ppm were produced. A verification test using optimum synthesis parameters also indicated insignificant (*p* > 0.05) differences between the values of predicted and experimental of λ_max_ and concentration of the fabricated Ag NPs and verified the suitability of the models.

### 2.3. Physico-Chemical Characteristics of the Synthesized Ag NPs at Obtained Optimum Conditions

**Figure 6:**
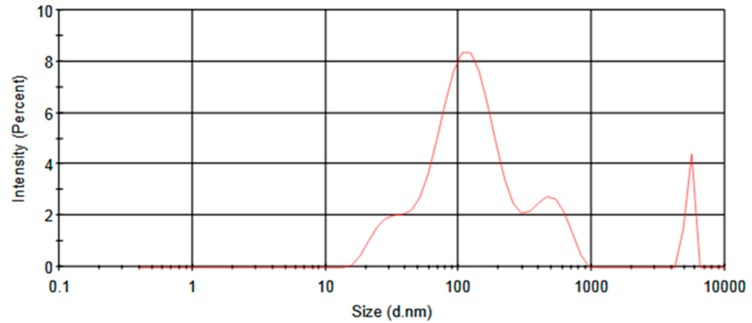
Particle size distribution of synthesized Ag NPs at obtained optimum synthesis conditions using *J. regia* leaf extract.

Formation of Ag NPs using *J. regia* leaf extract at obtained optimum conditions was confirmed by changes in the color of the mixture solution. Dynamic light scattering (DLS) analysis also indicated that the synthesized Ag NPs had particle size, polydispersity index (PDI), and zeta potential values of 168 nm, 0.419 and −15.6 mV, respectively. The particle size distributions (PSD) of the sample are also shown in Figure 6. 

### 2.4. Antibacterial Activity 

**Figure 7:**
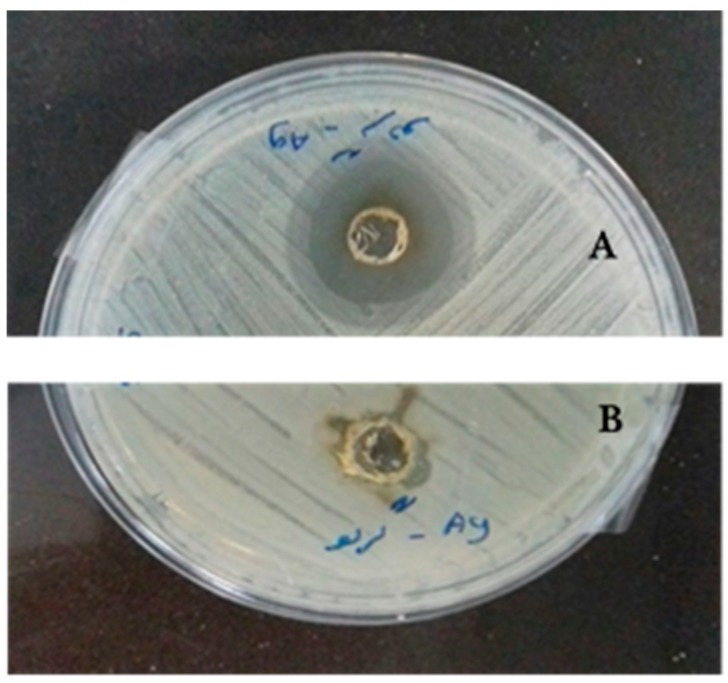
Created zones of inhibition with *S. aureus* (**A**) and *E. coli* (**B**) incubated at 37 °C for 24 h for synthesized Ag NPs using *J. regia* leaf extract.

The antibacterial activity of synthesized Ag NPs on the growth of Gram-positive (*S. aureus*) and Gram-negative (*E. coli)* bacteria during incubation indicated that the diameter of the clear zone for synthesized Ag NPs in the plate containing *S. aureus* and *E. coli* were 16 and 10 mm, respectively (Figure 7). The results also indicated that the mean diameter of formed clear zone around the Ampicillin disc in the plates containing *S. aureus* and *E. coli* were 35 and 33 mm, respectively.

The obtained results indicated that the fabricated Ag NPs had higher antibacterial activity against Gram-positive bacteria compared to the Gram-negative bacteria. The obtained results were in agreement with findings of Ahmadi et al., Mohammadlu et al., and Torabfam and Jafarizadeh-Malmiri [8,9,10]. The main reason behind the bactericidal activity of the Ag NPs against the bacteria strains is related to their effects on the permeability of the cell wall and membrane. In fact, the released silver ions (Ag^+^) from the Ag NPs were attached into the anionic groups of the cell wall, such as polycyclic aromatic hydrocarbon and teichoic acids. They also formed polyelectrolyte complexes, which limited the transference of nutrients and provided metabolites into and outside the cell [9]. Furthermore, caffeoylquinic acid is the main phenylpropanoids in *J. regia* leaf extract, which has various bioactivities such as antioxidant, antibacterial, anticancer, antihistamic, and other biological effects. The direct antimicrobial activity of caffeoylquinic acid implies an array of possibilities, including effects in the cell envelope [13].

## 3. Materials and Methods

### 3.1. Materials 

*J. regia* leaves were picked from walnut trees in Tabriz, Iran. AgNO_3_, as silver salt, was bought from Dr. Mojallali (Dr. Mojallali Chemical Complex Co., Tehran, Iran). Standard solution of Ag NPs (with particle size of 10 nm and concentration of 1000 ppm) was purchased from Tecnan-Nanomat (Spain). *Escherichia coli* (PTCC 1270) and *Staphylococcus aureus* (PTCC 1112) were attained from microbial Persian-type culture collection (PTCC, Tehran, Iran). There is no ATCC (American type culture collection) number for *E. coli* and this bacteria is clinical isolate. However, the ATCC number of *S. aureus* is 6538. Nutrient agar was bought from Biolife (Biolife Co., Milan, Italy). 

### 3.2. Preparation of J. regia Leaf Extract 

The *J. regia* leaf was washed, dried (in dark room), powdered, and 1 g of the prepared powder was added into 100 mL of boiling distilled water for 5 min. After cooling the solution, it was filtered (Whatman No. 1 filter paper) using a Buchner funnel under vacuum pressure and the clear walnut leaf extract was stored at a cold temperature (4 °C).

### 3.3. Ag NPs Synthesis Using J. regia Leaf Extract 

The Ag NPs solution was obtained by a domestic microwave-assisted synthetic approach. AgNO_3_ solution (1 mM) was made by dissolving 0.017 g of its powder in 100 mL of deionized double-distilled water. In a typical synthesis, different amounts of AgNO_3_ solution (15–25 mL) were mixed with different amounts of *J. regia* leaf extract (0.1–0.9 mL) and the mixture solutions were put into a microwave oven (MG-2312W, LG Co., Seoul, Korea) at a constant power of 800 W and microwave exposure time (180 s). 

### 3.4. Physico-Chemical Assay

#### 3.4.1. Fourier Transform-Infrared (FT-IR) Spectra Analysis 

In order to identify the possible reducing and stabilizing biomolecules of *J. regia* leaf extract, FT-IR measurements were carried out. The FT-IR spectrum of the extract was recorded on a Bruker Tensor27 spectrometer (Bruker Co., Karlsruhe, Germany) using KBr pellets in the 4000–400 cm^−1^ region.

#### 3.4.2. Surface Plasmon Resonance 

Ag NPs, due to their SPR, have a strong absorption of light, which is shown as broad emission peaks (λ_max_) in the wavelength ranging from 380 to 450 nm [8,9,10]. Therefore, formation of Ag NPs using *J. regia* leaf extract can be confirmed by the absorption spectrum of the mixture solutions containing fabricated Ag NPs by using a Jenway UV-Vis spectrophotometer 6705 (Cole-Parmer Co., Staffordshire, UK). Furthermore, by preparing several serial dilute Ag NPs solutions (10–1000 ppm) and establishing standard curves based on the defined concentrations of the Ag NPs solutions and their absorbance unit values, it is possible to determine the concentration of the formed Ag NPs using *J. regia* leaf extract [10]. 

#### 3.4.3. Particle Size, Particle Size Distribution, Polydispersity Index and Zeta Potential of the Synthesized Ag NPs

In order to measure values of the mean particle size (nm), PDI (ranging 0–1) and zeta potential (mV) of the fabricated Ag NPs and their PSD, a DLS particle size analyzer (Nanotrac Wave, Microtrac, Montgomeryville, PA, USA) was utilized. The DLS technique scatters a laser light beam at the surface of dispersed NPs, which results in the detection of the backscattered light. PDI is a dimensionless value which shows that the uniformity of the synthesized NPs and their surface electric charge are related to the PDI and zeta potential values of the synthesized NPs [14,15]. 

### 3.5. Antibacterial Assay 

Bactericidal activity of the formed Ag NPs was evaluated using the well diffusion method. For this reason, bacterial suspensions containing 1.5 × 10^8^ colony-forming units of bacteria was prepared based on a 0.5 McFarland standard and 0.1 mL of that amount was spread on the surface of solid nutrient agar in the plates and a hole, 5 mm in diameter, was made in the solid agar. 10 µL of the produced Ag NPs solution was then poured into the created well and put in incubator at 37 °C for 24 h. The antibacterial activity of the synthesized Ag NPs correlated to the diameter of created clear zones around the holes. An Ampicillin disc 5mm in diameter (Oxoid, 10 µg/disc) was used as a positive control for both Gram-positive and Gram-negative bacteria strains and the antibacterial activity of the synthesized Ag NPs was compared to the Ampicillin.

### 3.6. Experimental Design, Statistical Analysis and Optimization Procedure

The experiment was planned using a central composite design (CCD) and response surface methodology (RSM) was used to evaluate the effects of two independent parameters, namely amount of leaf extract (X_1_) and amount of AgNO_3_ solution (X_2_), on the prepared Ag NPs. The studied response variables were broad emission peak (λ_max_) (Y_1_, nm) and concentration (Y_2_, ppm) of the synthesized Ag NPs. As clearly observed in Table 1, thirteen experimental treatments were assigned with five different levels for each independent parameter using Minitab software (v.16 statistical package, Minitab Inc., Pennsylvania State, PA, USA). In order to correlate the λ_max_ (Y_1_) and concentration (Y_2_) of the synthesized Ag NPs to the studied synthesis variables, a second order polynomial equation (Equation (1)) was used. Where β_0_ is a constant, β_1_, β_11_, and β_12_ correspond to the linear, quadratic and interaction effects, respectively [16]. The suitability of the model was studied, accounting for the coefficient of determination (R^2^) and adjusted coefficient of determination (R^2^-adj) [17]. Analysis of variance (ANOVA) was also used to provide the significance determinations of the resulted models in terms of *p*-value. Small *p*-values (lower than 0.05) were considered as statistically significant [18].

Y = β_0_*+* β_1_X_1_ + β_2_X_2_ + β_11_X_1_^2^ + β_22_X_2_^2^ + β_12_X_1_X_2_ (1)

Numerical optimization was carried out to determine exact amounts of silver salt and *J. regia* leaf extract in the Ag NPs synthesis to produce NPs with minimum λ_max_ (particle size) and maximum concentration. Graphical optimization was also used to better visualize the effects of the synthesis parameters on the response variables (λ_max_ and concentration) [19]. Suitability and accuracy of the generated models in predicting the response variables in the defined range of the Ag NPs synthesis parameters were validated by synthesis of Ag NPs using obtained optimum synthesized conditions and comparison of the experimental values of the response variables and their predicted values [20].

## 4. Conclusions

Fabricated Ag NPs using a rapid, one-step green approach, based on microwave irradiation showed desirable physico-chemical properties with antibacterial activity against *E. coli* and *S. aureus*. However, their antibacterial activity was significantly (*p* < 0.05) higher toward Gram-positive bacteria strains. The synthesized Ag NPs using the rapid developed synthesis method can be used as a favorable antibacterial agent in different areas such as medicine and food packaging.

## Figures and Tables

**Table 1:** Experimental runs according to the central composite design (CCD) and response variables for synthesis of Ag NPs.

Sample No.	Amount of Leaf Extract (mL)	Amount of Silver Salt (mL)	λ_max_ (nm)	Concentration (ppm)		
			Exp	Pre	Exp	Pre
1	0.10	20.00	425	424.896	104.813	101.702
2	0.78	16.46	429	429.104	100.003	97.802
3	0.78	23.53	424	424.00	93.503	96.614
4	0.50	15.00	427	426.854	72.443	74.643
5	0.50	25.00	*	*	48.393	43.081
6	0.50	20.00	426	427.250	56.453	61.835
7	0.50	20.00	426	427.250	56.453	61.835
8	0.217	23.53	426	426.043	50.343	55.654
9	0.50	20.00	428	427.250	66.073	61.835
10	0.90	20.00	427	426.957	*	*
11	0.217	16.46	424	424.146	*	*
12	0.50	20.00	429	427.250	66.723	61.835
13	0.50	20.00	*	*	63.473	61.835

Exp, Experimental values of studied responses; Pre, Predicted values of studied responses. *, Out of range.

**Table 2:** Regression coefficients, R^2^, R^2^-adj, and probability values for the fitted models.

Regression Coefficient	λ_max_ (nm)	Concentration (ppm)
β_0_ (constant)	386.44	249.7
β_1_ (main effect)	−45,084	−513.04
β_2_ (main effect)	3.09	−3.68
β_11_ (quadratic effect)	−8.27	336.8
β_22_ (quadratic effect)	−0.061	−0.119
β_12_ (interaction effect)	−1.75	10.56
R^2^	77.81%	95.25%
R^2^-adj	85.62%	90.49%
Lack of fit (*p*-value)	0.985	0.142

β_0_ is a constant, β_1_, β_11_ and β_12_ are the linear, quadratic, and interaction coefficients of the quadratic polynomial equation, respectively.

**Table 3:** *p*-Values of the regression coefficients in the obtained models.

Main Effects	Main Effects		Quadratic Effects		Interacted Effect
	X_1_	X_2_	X_11_	X_22_	X_1_ X_2_
λ_max_ (nm) (*p*-value)	0.003	0.693	0.00	0.580	0.045
Concentration (ppm) (*p*-value)	0.018	0.153	0.224	0.247	0.030

## References

[1] Mohammadlu M., Jafarizadeh-Malmiri H., Maghsoudi H. (2016). A review on green silver nanoparticles based on plants: Synthesis, potential applications and eco-friendly approach. Int. Food Res. J..

[2] Kon K., Rai M. (2013). Metallic nanoparticles: Mechanism of antibacterial action and influencing factors. J. Comp. Clin. Pathol. Res..

[3] Zhou Y., Kong Y., Kundu S., Cirillo J.D., Liang H. (2012). Antibacterial activities of gold and silver nanoparticles against *Escherichia coli* and *Bacillus calmette-Guérin*. J. Nanobiotechnol..

[4] Ingale A.G., Chaudhari A. (2013). Biogenic synthesis of nanoparticles and potential applications: An eco-friendly approach. J. Nanomed. Nanotechnol..

[5] Hebbalalu D., Lalley J., Nadagouda M.N., Varma R.S. (2013). Greener techniques for the synthesis of silver nanoparticles using plant extracts, enzymes, bacteria, biodegradable polymers, and microwaves. ACS Sustain. Chem. Eng..

[6] Vamanu E., Ene M., Bita B., Ionescu C., Craciun L., Sarbu I. (2018). In vitro human microbiota response to exposure to silver nanoparticles biosynthesized with mushroom extract. Nutrients.

[7] Zhang Y., Cheng X., Zhang Y., Xue X., Fu Y. (2013). Biosynthesis of silver nanoparticles at room temperature using aqueous Aloe leaf extract and antibacterial properties. Colloids Surf. A Physicochem. Eng. Asp..

[8] Mohammadlu M., Jafarizadeh-Malmiri H., Maghsoudi H. (2017). Hydrothermal green silver nanoparticles synthesis using *Pelargonium*/*Geranium* leaf extract and evaluation of their antifungal activity. Green Process. Synth..

[9] Ahmadi O., Jafarizadeh-Malmiri H., Jodeiri N. (2018). Eco-friendly microwave enhanced green silver nanoparticles synthesis using Aloe vera leaf extract and their physico-chemical and antibacterial studies. Green Process. Synth..

[10] Torabfam M., Jafarizadeh-Malmiri H. (2017). Microwave–enhanced silver nanoparticles synthesis using chitosan biopolymer–Optimization of the process conditions and evaluation of their characteristics. Green Process. Synth..

[11] Jaiswal B.S., Tailang M. (2017). *Juglans regia*: A review of its traditional uses phytochemistry and pharmacology. Indo Am. J. Pharm. Res..

[12] Amaral J.S., Seabra R.M., Andrade P.B. (2004). Phenolic profile in the quality control of walnut (*Juglans regia* L.) leaf. Food Chem..

[13] Pereira J.A., Oliveira I., Sousa A. (2007). Walnut (*Juglans regia* L.) leaf: Phenolic compounds, antimicrobial activity and antioxidant potential of different cultivars. Food Chem. Toxicol..

[14] Eskandari-Nojedehi M., Jafarizadeh-Malmiri H., Rahbar-Shahrouzi J. (2016). Optimization of processing parameters in green synthesis of gold nanoparticles using microwave and edible mushroom (*Agaricus bisporus*) extract and evaluation of their antibacterial activity. Nanotechnol. Rev..

[15] Eskandari-Nojedehi M., Jafarizadeh-Malmiri H., Jafarizad A. (2018). Microwave accelerated green synthesis of gold nanoparticles using gum Arabic and their physico-chemical properties assessments. Z. Phys. Chem..

[16] Amirkhani L., Moghaddas J., Jafarizadeh-Malmiri H. (2016). *Candida rugosa* lipase immobilization on magnetic silica aerogel nanodispersion. RSC Adv..

[17] Anarjan N., Jaberi N., Yeganeh-Zare S., Banafshehchin E., Rahimirad A., Jafarizadeh-Malmiri H. (2014). Optimization of mixing parameters for α-tocopherol nanodispersions prepared using solvent displacement method. J. Am. Oil Chem. Soc..

[18] Eskandari-Nojedehi M., Jafarizadeh-Malmiri H., Rahbar-Shahrouzi J. (2018). Hydrothermal biosynthesis of gold nanoparticle using mushroom (*Agaricus bisporous*) extract: Physico-chemical characteristics and antifungal activity studies. Green Process. Synth..

[19] Anarjan N., Jafarizadeh-Malmiri H., Nehdi I.A., Sbihi H.M., Al-Resayes S.I., Tan C.P. (2015). Effects of homogenization process parameters on physicochemical properties of astaxanthin nanodispersions prepared using a solvent-diffusion technique. Int. J. Nanomed..

[20] Ahdno H., Jafarizadeh-Malmiri H. (2017). Development of a sequenced enzymatically pre-treatment and filter pre-coating process to clarify date syrup. Food Bioprod. Process..

